# Structure–Property Relationships of Polysiloxane Networks Containing Linear, Cyclic, and Tetrakis Units

**DOI:** 10.1021/acsomega.6c00169

**Published:** 2026-04-03

**Authors:** Virginia C. Mullins, Davide L. Simone, Jeffrey S. Wiggins, William Jacob Monzel

**Affiliations:** † 5104 University of Southern Mississippi, 118 College Drive, Hattiesburg, Mississippi 39406, United States; ‡ BlueHalo, an AV Inc. Company, 4401 Dayton Xenia Road, Dayton, Ohio 45432, United States; § 33319 Air Force Research Laboratory, 2941 Hobson Way, WPAFB, Ohio 45433, United States

## Abstract

Polysiloxanes are a highly relevant family of polymers capable of producing glassy networks with excellent thermal stability; however, studies on the impact of backbone substitution are limited. Formulations comprising cyclic and linear polysiloxanes were prepared, and cross-linked polymer networks were subsequently formed via hydrosilylation. Optically transparent networks with varying degrees of rigidity were obtained, and the effects of the cyclic-to-linear ratio on the physical properties of the resulting networks were investigated. We demonstrate the preparation of cross-linked polysiloxanes and report thermal stability and transitions, thermal expansion behavior, and mechanical properties. Char yields ranged from 45.7 to 85.0% in nitrogen with no significant reductions under air, indicating the potential for high-temperature applications. Mechanical testing revealed that the incorporation of linear siloxane segments increased network flexibility, while cyclic siloxane structures resulted in rigid networks with lower thermal expansion. These results establish a direct relationship between siloxane precursor structure and the thermomechanical properties of cross-linked polysiloxane networks, providing insight into the design of polymer materials with tunable mechanical and thermal performance.

## Graphical abstract

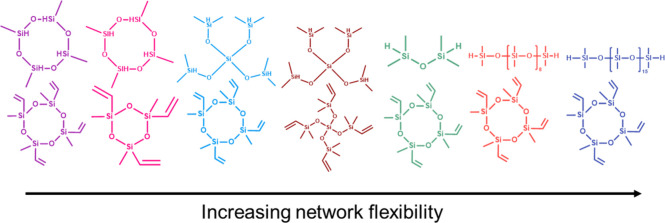

## Introduction

Siloxane polymers are high-performance materials that exhibit excellent thermal and chemical stability.^1,2^ Polysiloxanes are composed of a large family of diverse backbone structures and side groups and have thus been utilized in many fields, including adhesives,^3^ coatings,^4^ and redox catalysis.^5^ Within polysiloxanes, linear siloxanes have proven useful in applications requiring optical transparency,^6^ low surface tension,^7^ and flame retardancy.^8^ However, flexible, linear siloxanes undergo dramatic degradation under both oxidative and inert environments.^9^ Under inert conditions, linear siloxanes undergo bond exchange reactions, including chain rearrangement and unzipping mechanisms, resulting in a reduction in molecular weight and the formation of volatile cyclosiloxanes.^10^ In contrast, oxidative environments result in cross-linking via organic functional groups with the removal of carbon dioxide and water.^11^ Both degradation mechanisms are inhibited by steric hindrance and can be suppressed by the presence of aryl substituents in the polymer backbone.^12^ If the backbone structure avoids linear siloxane segments and instead already contains cyclic rings, then degradation by unzipping and the formation of cyclics is precluded, which enhances thermal stability. Thus, cyclosiloxanes are of particular interest for high-temperature applications due to their increased resistance to degradation.^13,14^

Cyclosiloxane networks exhibit improved mechanical strength due to extensive branching during polymerization.^3^ Much recent work on cyclosiloxanes has focused on networks formed from 1,3,5,7-tetravinyl-1,3,5,7-tetramethylcyclotetrasiloxane (D_4_^Vi^) and 1,3,5,7-tetramethylcyclotetrasiloxane (D_4_^H^).^16^ These materials exhibit intriguing characteristics such as optical transparency,^17^ hardness,^18^ and thermal stability^16^ and have proven to be useful in applications such as nanoimprint lithography,^19^ hydrophobic dielectrics,^20^ and polymer-derived ceramics.^21^ However, practical applications of these networks are limited by their inherent brittleness, and necessary structure–property relationships remain underdeveloped. Existing strategies to improve the physical properties of D_4_^Vi^:D_4_^H^ networks rely on incorporating other siloxane structures^18^ or nanoparticles.^17^ A recent study focused on high-temperature adhesive materials found that the molar ratio of D_4_^Vi^ to D_4_^H^ could be varied to optimize performance.^3^ Additionally, Nyczyk and co-workers made preceramic polymers using D_4_^Vi^ and a variety of hydrogensiloxanes, including D_4_^H^, and analyzed the ceramic yield, composition, and morphology.^21^ Notably, by designing networks with a mixture of cyclic and linear components, tailorable physical properties can be achieved. For example, cyclo-linear siloxanes with high phenyl content and refractive index were developed for optoelectronic applications.^15^ However, limited research has described the structure–property relationships of polysiloxane networks with varying levels of rigidity. Appropriate incorporation of flexible monomers is key to improving the toughness of polysiloxane networks while maintaining their thermomechanical stability. The siloxane units in the prepolymer will determine the properties of the resulting cross-linked network and subsequently the ceramic properties after pyrolysis.^22,23^ Herein, we demonstrate the preparation of siloxane networks with varying degrees of rigidity and explore the impacts of network rigidity on the mechanical properties and thermal degradation behavior.

## Results and Discussion

### Network Formulation

Figure 1 presents the resin chemical structures and nomenclature. Seven resins were formulated with each formulation combining a Si–H functionalized monomer with a Si-vinyl functionalized monomer at a molar ratio of 1:1. In each case, the Si-vinyl functionalized monomer was first combined with 50 ppm Karstedt’s catalyst and then added to the Si–H functionalized monomer to prevent excessive exothermicity. The formulations are as follows: D_4_^H^:D_4_^Vi^, Q­(M^H^)_4_:D_4_^Vi^, M^H^M^H^:D_4_^Vi^, H08:D_4_^Vi^, H15:D_4_^Vi^, D_4_^H^:D_3_^Vi^, and Q­(M^H^)_4_:Q­(M^Vi^)_4_. Samples were cured in air with a three-step process: 24 h at room temperature, 4 h at 70 °C, and 4 h at 150 °C.

**Figure 1:**
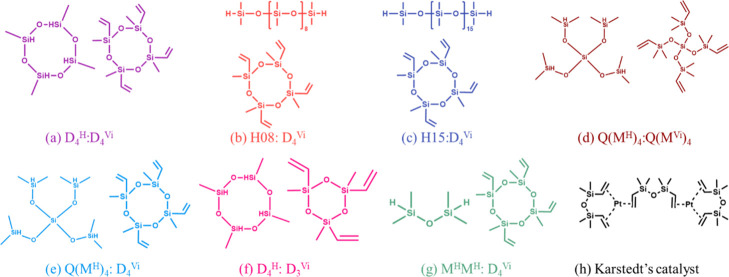
Formulation structures.

### Polysiloxane Network Characterization

Thermomechanical analysis (TMA) was performed to measure dimensional changes as a function of temperature and to calculate the coefficient of thermal expansion (CTE). Thermograms are provided in Figure 2, and extracted values are presented in Table 1. In comparison to pure PDMS (CTE = 354 μm/m °C),^24^ the materials in this study exhibited CTE values in the range of 100–302 μm/m °C. CTE exhibited a strong dependence on network architecture and systematically increased as the prevalence and length of flexible linear siloxane segments increased and cross-link density and ring structures decreased. The highly flexible H15:D_4_^Vi^, which had the longest linear segment, proved to have the highest CTE value (302 μm/m °C). In contrast, the rigid D_4_^H^:D_4_^Vi^ displayed the lowest CTE (100 μm/m °C) as a result of its highly cyclic nature.

**Figure 2:**
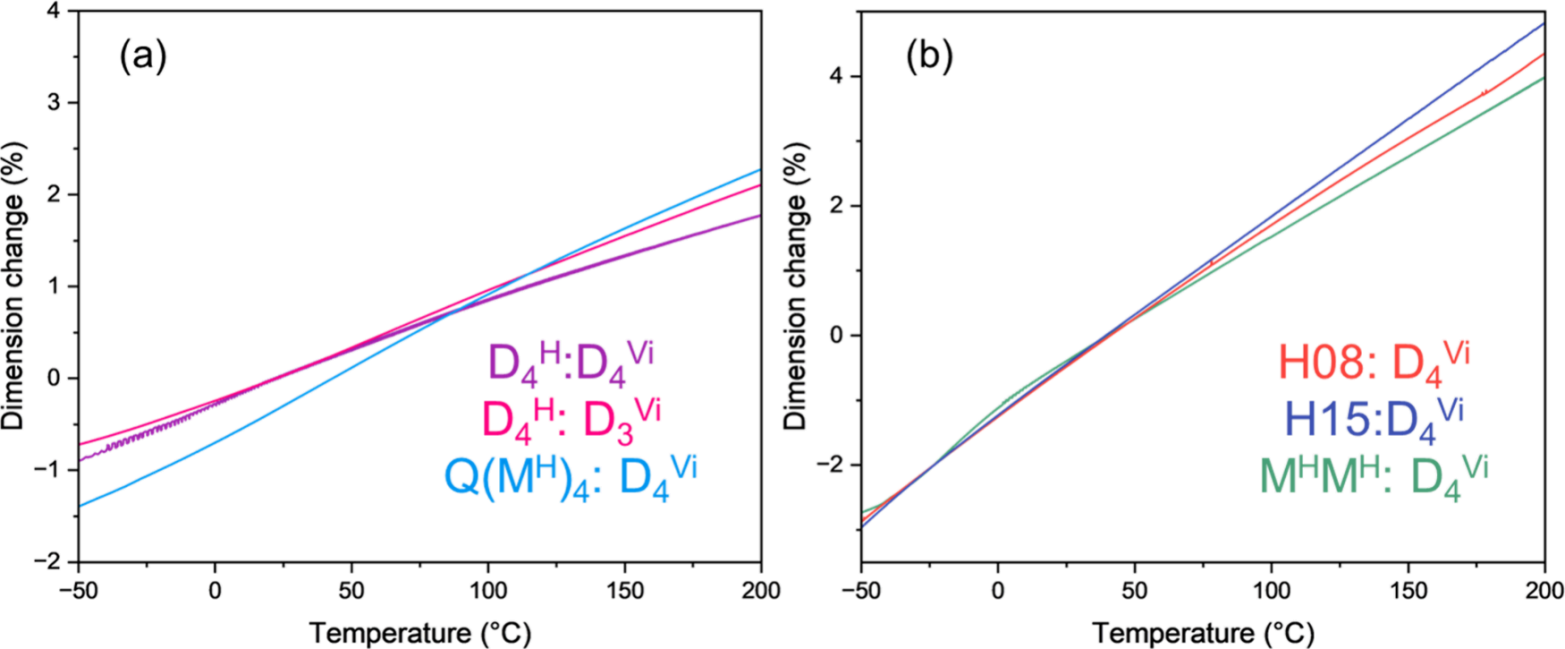
TMA thermograms showing thermal expansion of rigid networks (a) and flexible networks (b).

**Table 1:** CTE Values from TMA

sample	rubbery CTE (μm/m °C)
D_4_^H^:D_4_^Vi^	100
D_4_^H^:D_3_^Vi^	108
Q(M^H^)_4_:D_4_^Vi^	131
M^H^M^H^:D_4_^Vi^	248
H08:D_4_^Vi^	274
H15:D_4_^Vi^	302

This trend is consistent with greater thermally induced expansion in networks with longer linear siloxane chains due to increased free volume and segmental mobility.^25^ In contrast, volumetric expansion is suppressed in rigid networks with increased cyclic content due to topological constraints that limit chain mobility.^13,26^ These findings indicate that CTE can be tuned as a function of cyclic ring concentration.^25,26^ Although subtle inflections indicating *T*_g_ are seen in the curves for M^H^M^H^:D_4_^Vi^ and Q­(M^H^)_4_:D_4_^Vi^, inflection points were not noticeable in any other samples. This behavior suggests that the glass transition temperatures of these networks are below the measurable range of the instrument, indicating that the transitions are inherently outside the accessible measurement window rather than absent. This limitation also applied to differential scanning calorimetry (DSC), where a noticeable baseline shift was observed only for M^H^M^H^:D_4_^Vi^ (Figure S1).

To better understand the phase transition behavior of these materials, dynamic mechanical analysis (DMA) was employed. Glass transition regions and storage moduli of rigid networks (D_4_^H^:D_4_^Vi^, D_4_^H^:D_3_^Vi^, and Q­(M^H^)_4_:D_4_^Vi^) were investigated using DMA, resulting in thermograms presented in Figure 3. Notably, flexible formulations (M^H^M^H^:D_4_^Vi^, H08:D_4_^Vi^, and H15:D_4_^Vi^) were too soft to make suitable samples for 3-point bending tests, consistent with their high chain mobility perceived by TMA. A glassy plateau of the storage modulus is not observed in the measurement temperature range, indicating that *T*_g_ values by storage modulus are below −50 °C. tan δ peaks are observed at approximately −25 °C (D_4_^H^:D_3_^Vi^), −6 °C (D_4_^H^:D_4_^Vi^), and 10 °C (Q­(M^H^)_4_:D_4_^Vi^). The broad nature of the transitions is characteristic of highly cross-linked networks and suggests a distribution of relaxation processes caused by network heterogeneity.^3,27^

**Figure 3:**
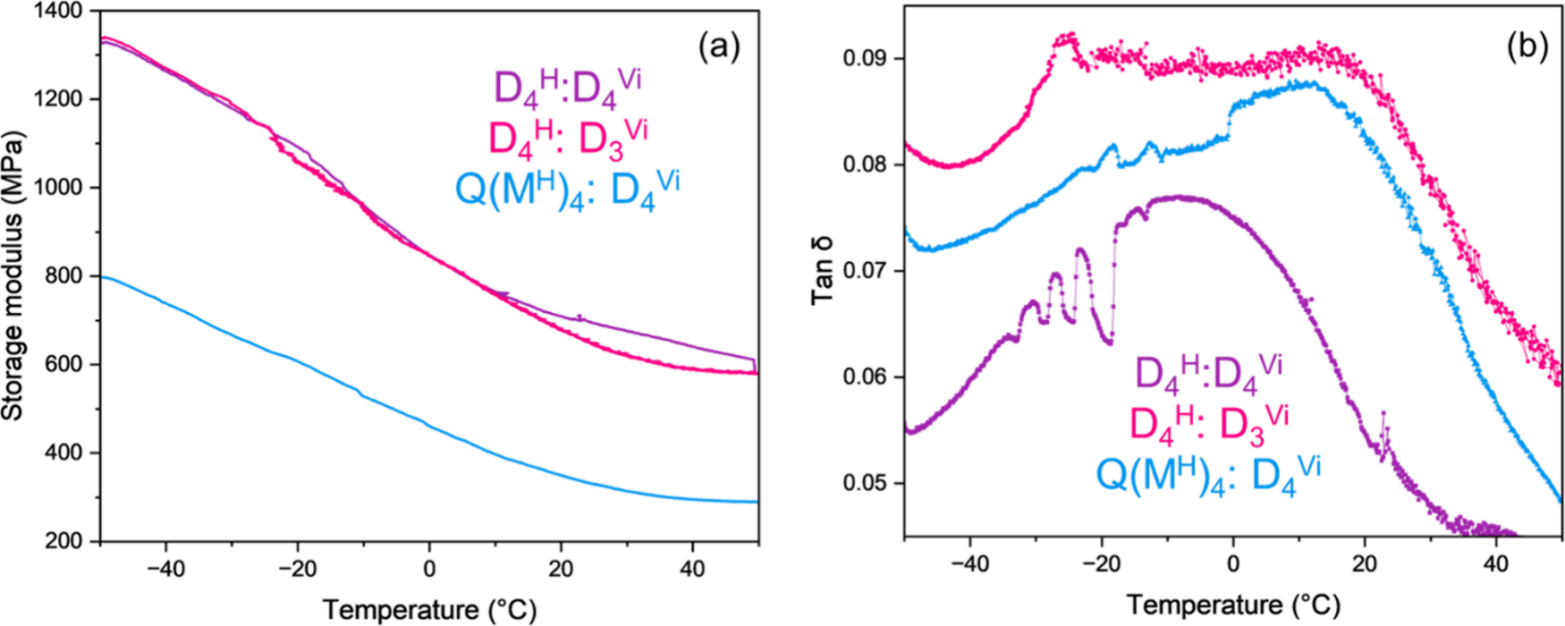
DMA results: storage modulus (a) and tan δ (b).

### Degradation Behavior of Polysiloxane Networks

To investigate the important thermal transformations and evolved gas species of our polysiloxane networks during degradation, thermogravimetric analysis (TGA) coupled with Fourier transform infrared spectroscopy (FTIR) was used. Char yield (mass remaining at 1000 °C) under nitrogen atmosphere ranges from 45.67% (Q­(M^H^)_4_:Q­(M^Vi^)_4_) to 84.98% (D_4_^H^:D_4_^Vi^) as shown in Figure 4, demonstrating a strong dependence of thermal stability on network architecture. The relatively low yield observed for Q­(MH)_4_:Q­(MVi)_4_ is attributed to rapid exothermic curing at room temperature, which led to foaming and likely introduced additional porosity into the network. A multistep process with partial additions of hydride to vinyl could potentially mitigate the exotherm in the future. D_4_^H^:D_4_^Vi^ exhibits mass loss events at approximately 410, 584, and 691 °C (Table 2 and Figure 5). TGA-FTIR indicates that methane (3016, 1305 cm^–1^)^28^ and ethylene (950 cm^–1^)^29^ are the primary degradation products. Ethylene evolves primarily at 584 °C and is attributed to the degradation of ethylene bridges and any residual vinyl groups.^30^ Methane continued to evolve at 691 °C and was primarily ascribed to cleavage of methyl functional groups pendant to silicon.^22^ Minimal mass loss before 410 °C suggests that few vinyl groups remain, as well as high stability of Si–C bonds at lower temperatures.^22^ D_4_^H^:D_3_^Vi^ exhibits very similar thermograms to D_4_^H^:D_4_^Vi^ under both nitrogen and air, aside from a reduction in noticeable degradation (357 °C), indicating that the change in ring size from 8-membered to 6-membered has little effect on thermal stability. Cyclic siloxanes are thermodynamically favored, and it is unlikely that significant mass loss is caused by the rearrangement of rings or cleavage of ring bonds.^31^ Additional FTIR of significant peaks for each network as a function of temperature is provided in Figures S2–S8.

**Figure 4:**
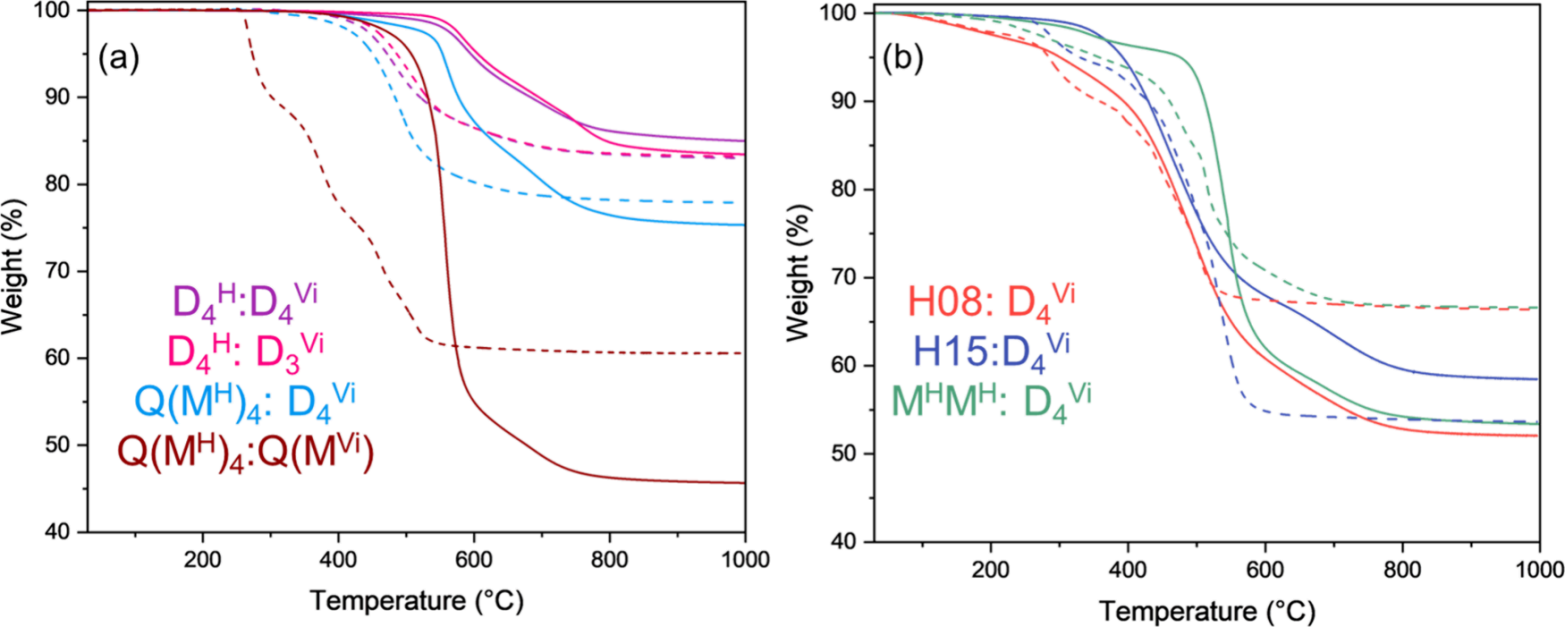
TGA thermograms of rigid networks (a) and flexible (b) under N_2_ (solid lines) and air (dashed lines).

**Table 2:** Mass Loss Data Collected from TGA

sample	*T*_d_ 5% (°C) N_2_	*T*_d_ 5% (°C) air	yield at 1000 °C (%) N_2_	yield at 1000 °C (%) air
D_3_^Vi^:D_4_^H^	605.4	485.8	83.4	83.2
D_4_^H^:D_4_^Vi^	595.0	471.6	85.0	83.0
Q(M^H^)_4_:D_4_^Vi^	553.3	450.6	75.3	77.9
Q(M^H^)_4_:Q(M^Vi^)_4_	507.0	299.0	45.7	59.3
H08:D_4_^Vi^	300.8	284.4	52.1	66.4
H15:D_4_^Vi^	392.0	329.5	58.5	53.7
M^H^M^H^:D_4_^Vi^	357.8	357.8	66.6	66.6

**Figure 5:**
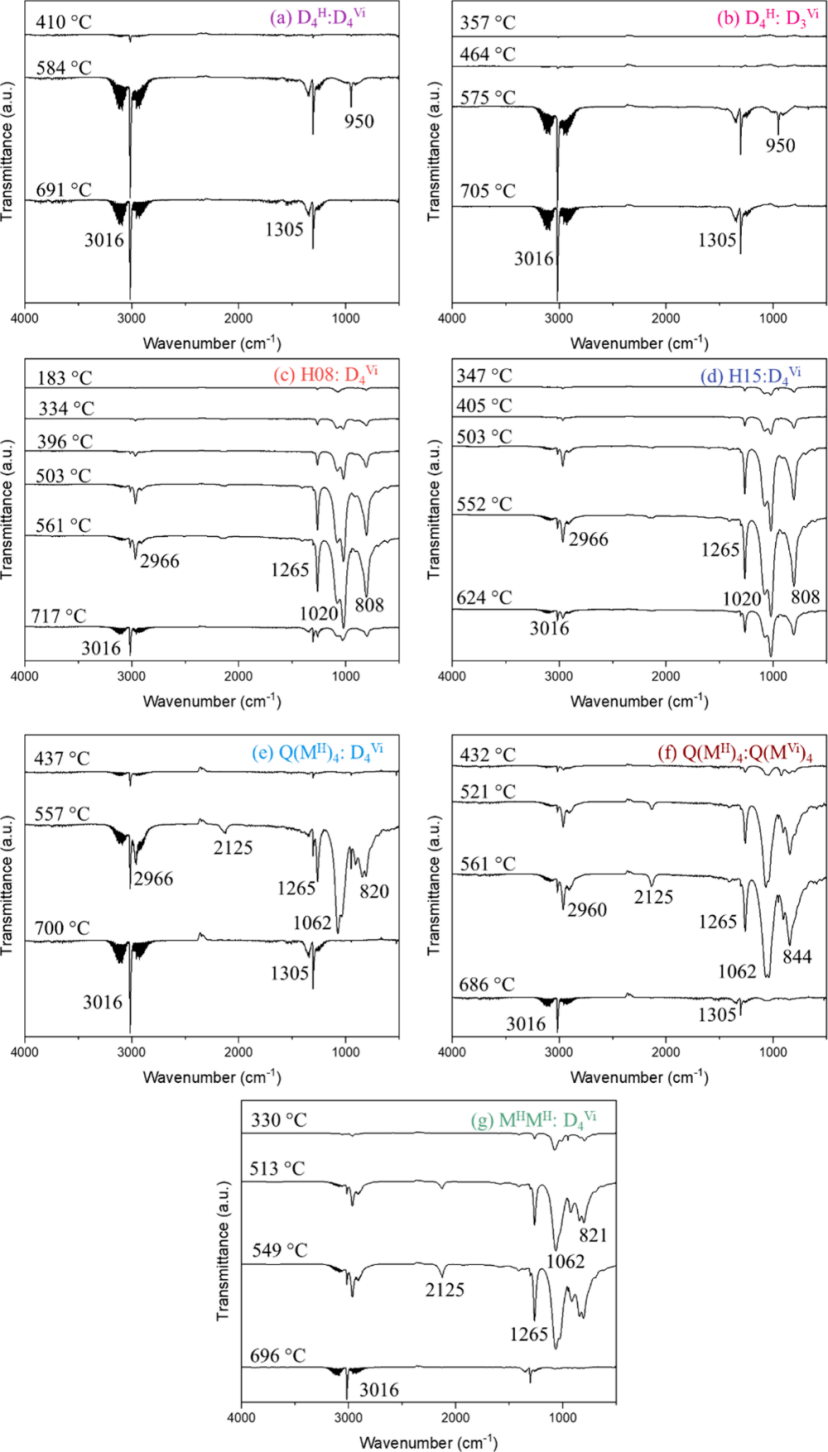
FTIR spectra of evolved gas products at peak degradation temperatures for (a) D_4_^H^:D_4_^Vi^, (b) D_4_^H^:D_3_^Vi^, (c) H08:D_4_^Vi^, (d) H15:D_4_^Vi^, (e) Q­(M^H^)_4_:D_4_^Vi^, (f) Q­(M^H^)_4_:Q­(M^Vi^)_4_, (g) M^H^M^H^:D_4_^Vi^.

All networks produce methane and ethylene as primary degradation products; however, systems with decreased cyclic content also exhibit evolution of silicon species. These products are attributed to rearrangement mechanisms, including backbiting or interchain exchange, producing products with smaller quantities of larger rings (D_5_, etc.).^6^ Above 300°, linear PDMS undergoes intrachain rearrangement to form a shorter PDMS chain and a volatile cyclic siloxane.^7,8^ Thus, M^H^M^H^:D_4_^Vi^, H08:D_4_^Vi^, and H15:D_4_^Vi^ all exhibit significantly lower char yields due to the additional decomposition mechanism via backbiting or interchain exchange. H08:D_4_^Vi^ displayed the lowest onset of degradation temperature, which is hypothesized to be caused by the presence of low molecular weight oligomers. H08:D_4_^Vi^ and H15:D_4_^Vi^ exhibit similar TGA-FTIR spectra and show peaks at 1265 and 808 cm^–1^ that are most likely due to Si-CH_3_ species as well as Si–O–Si peaks at 1020 cm^–1^.^9,10^ Additionally, Q­(M^H^)_4_:D_4_^Vi^, Q­(M^H^)_4_:Q­(M^Vi^)_4_, and M^H^M^H^:D_4_^Vi^ exhibit peaks at 2125 cm^–1^, which are attributed to Si–H.^11^

## Conclusions

The polymeric properties of seven siloxane networks with varying levels of flexibility were elucidated. Rigid networks (D_4_^H^:D_4_^Vi^, D_4_^H^:D_3_^Vi^, and Q­(M^H^)_4_:D_4_^Vi^) exhibited wide transition regions, as is common in highly cross-linked systems. CTE values decreased with increasing backbone rigidity and degree of cross-linking, with D_4_^H^:D_4_^Vi^ exhibiting the lowest CTE and H15:D_4_^Vi^ exhibiting the highest. While minimizing CTE is important to reduce thermal stresses during heating, the ability to tune CTE is essential for manufacturing complex systems to prevent interfacial stress concentrations caused by CTE mismatches. Exposure to air instead of nitrogen during TGA tests did not significantly reduce the yield at 1000 °C for all samples, although the initial degradation events typically occurred at lower temperatures due to the oxidation of ethylene bridges and methyl groups. Our results demonstrate the ability to tune the physical properties of high-performance siloxane matrices based on polymer backbone modulation. Future work should further investigate molecular structure variations to D_4_^H^ and D_4_^Vi^-based networks to reduce brittleness without significantly compromising the thermal and dimensional stability.

## Experimental Section

### Materials

1,3,5,7-Tetravinyl-1,3,5,7-tetramethylcyclotetrasiloxane (D_4_^Vi^), tetrakis­(dimethylsiloxy)­silane (Q­(M^H^)_4_), 1,3,5,7-tetramethylcyclotetrasiloxane (D_4_^H^), 1,3,5-trivinyl-1,3,5-trimethylcyclosiloxane (D_3_^Vi^), hydride terminated PDMS with 8 (H08) and 15 (H15) repeat units, tetrakis­[dimethyl­(vinyl)­silyl] orthosilicate (Q­(M^Vi^)_4_), Karstedt’s catalyst, and 1,1,3,3-tetramethyldisiloxane (M^H^M^H^) were purchased from Gelest Inc.

### Characterization

Differential scanning calorimetry (DSC) was performed using a TA Instruments Discovery 2500 under nitrogen atmosphere at a flow rate of 50 mL/min and a ramp rate of 5 °C/min. Thermomechanical analysis was conducted using a TA Instruments Discovery 450 with an expansion probe. Experiments were performed using a force of 0.05–0.5 N, a ramp rate of 5 °C/min, and a nitrogen flow rate of 50 mL/min. Thermal stability was measured using thermogravimetric analysis (TGA) on a TA Instruments Discovery 5500. Pieces of solid sample were heated to 1000 °C at a ramp rate of 10 °C/min under an atmosphere of either air or nitrogen at a flow rate of 50 mL/min. Dynamic mechanical analysis (DMA) was performed at 1 °C/min under flowing nitrogen using a TA Instruments Discovery Hybrid Rheometer (DHR) equipped with a 3-point bending fixture. Thermogravimetric analysis coupled with Fourier-transform infrared spectroscopy (TGA-FTIR) was carried out using a Netzsch TG 209F1 Libra ramping at 10 °C/min under 250 mL/min N_2_ and a Bruker INVENIO X equipped with a TGA-IR II module.

## Supplementary Material



## Notes

DSC ramps (Figure S1) and graphs of temperature vs significant IR peaks (Figures S2–S8) (PDF)
