# Supplementary material for: Structure–Property Relationships of Polysiloxane Networks Containing Linear, Cyclic, and Tetrakis Units
Source: ACS Omega. 2026 Apr 3;11(14):22262–7. doi: 10.1021/acsomega.6c00169 (PMC13084619; doi:10.1021/acsomega.6c00169)
Supplement: Supplementary file 1 [file ao6c00169_si_001.pdf]

Supplementary Information

Structure-Property Relationships of Polysiloxane Networks Containing Linear, Cyclic, and Tetrakis Units

Virginia C. Mullins[1,2], Davide L. Simone[3], Jeffrey S. Wiggins[1], and William Jacob Monzel[3]

1) University of Southern Mississippi, 118 College Drive, Hattiesburg, MS 39406
2) BlueHalo, an AV Inc. Company, 4401 Dayton Xenia Rd, Dayton, OH 45432
3) Air Force Research Laboratory, 2941 Hobson Way, WPAFB, OH 45433

virginia.mullins@usm.edu

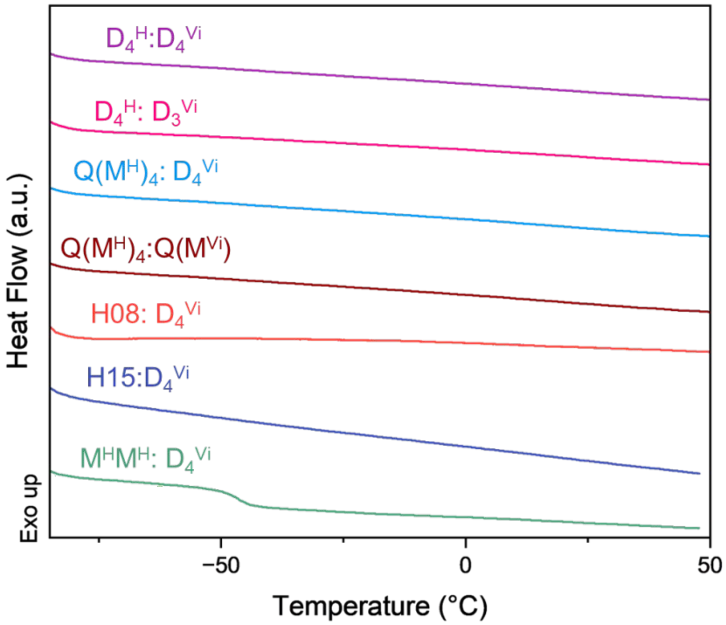

Figure S1. DSC ramps (10 °C/min)

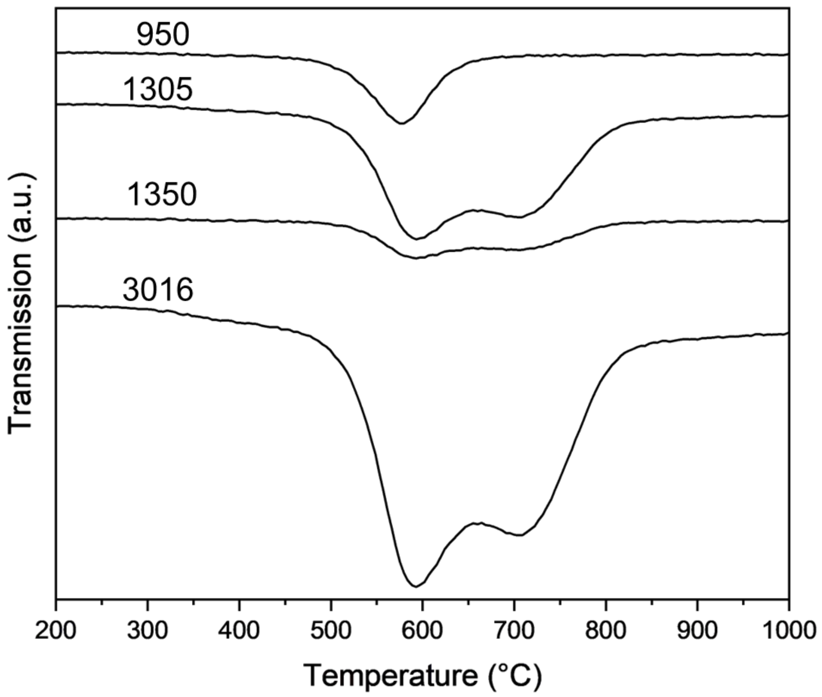

Figure S2. Temperature vs significant IR peaks (D_4^H:D_4^{Vi})

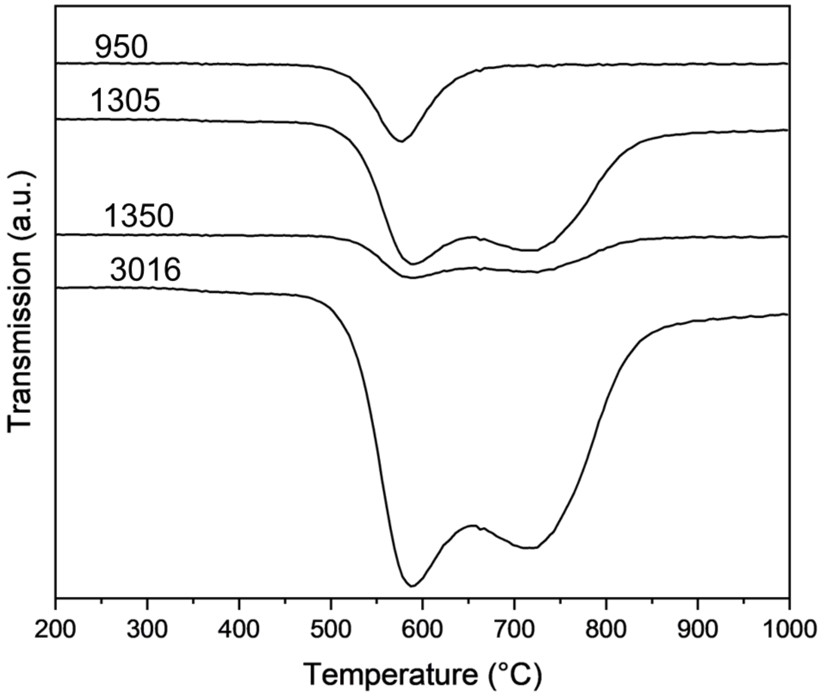

Figure S3. Temperature vs significant IR peaks (D_4^H:D_3^{Vi})

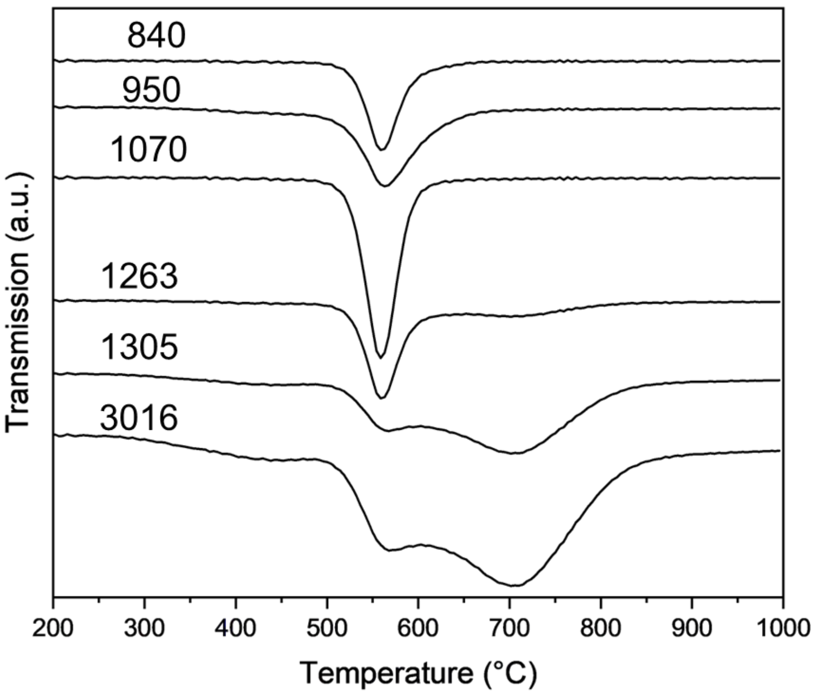

Figure S4. Temperature vs significant IR peaks ($Q(M^H)_4$: D_4^{Vi})

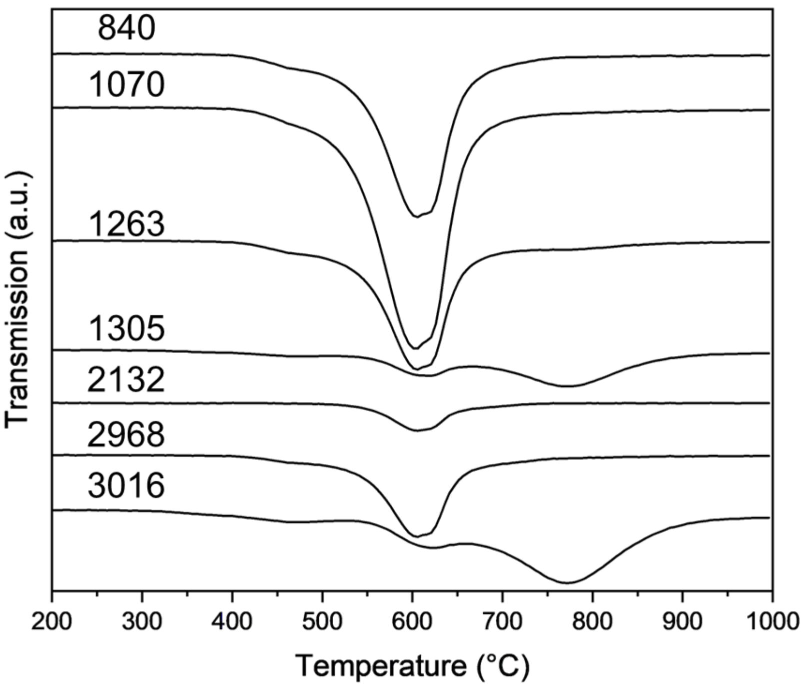

Figure S5. Temperature vs significant IR peaks ($Q(M^{H})_4$:$Q(M^{Vi})$)

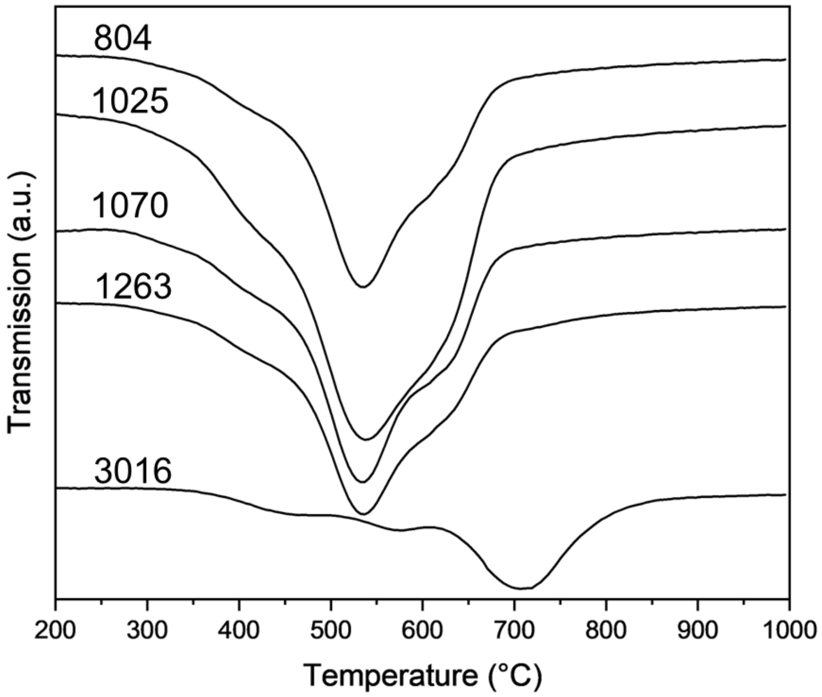

Figure S6. Temperature vs significant IR peaks (H08: D_4^{Vi})

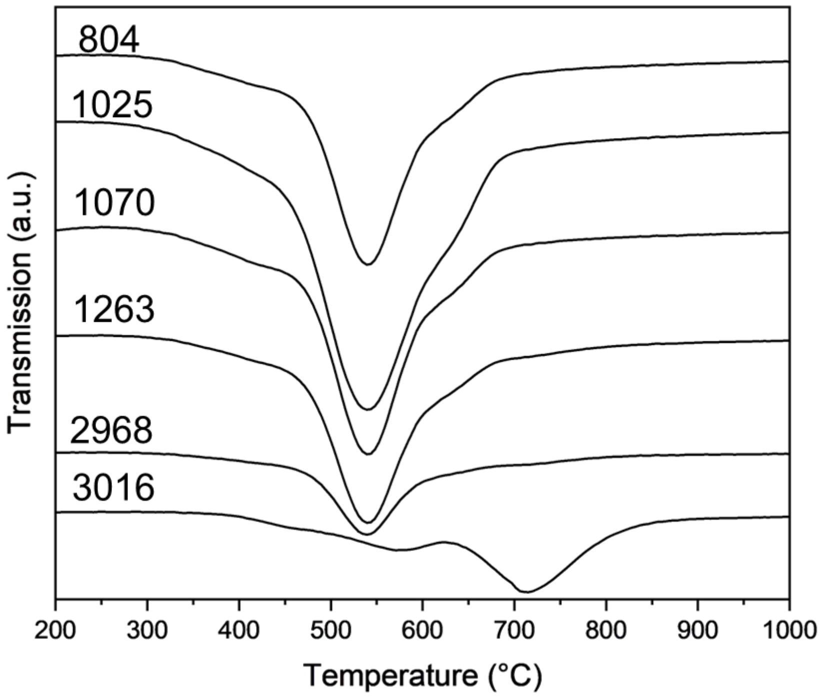

Figure S7. Temperature vs significant IR peaks (H15: D_4^{Vi})

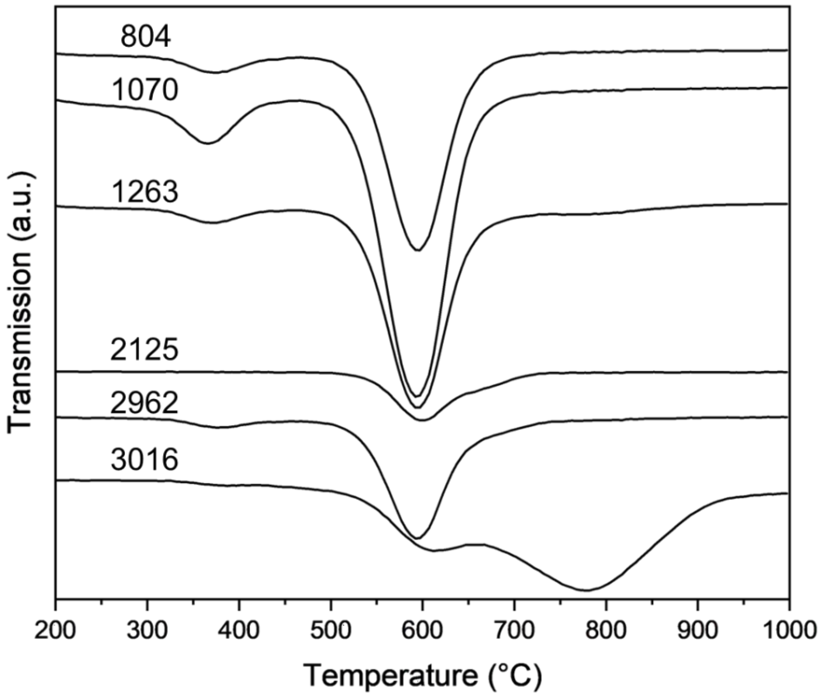

Figure S8. Temperature vs significant IR peaks (M^HM^H: D_4^{Vi})
